# Research Progress of Regulatory Cell Death in Coronary Microembolization

**DOI:** 10.7150/ijms.105295

**Published:** 2025-01-01

**Authors:** Chen Chang, Wan-Zhong Huang, Ru-Ping Cai, Li-Rong Mo, Qiang Wu, Qiang Su

**Affiliations:** 1 Department of Cardiology, The First Affiliated Hospital of Xi'an Medical University, Xi'an 710077, Shanxi, People's Republic of China.; 2 Department of Cardiology, Jiangbin Hospital of Guangxi Zhuang Autonomous Region, Nanning 530021, Guangxi, People's Republic of China.; 3 Department of Cardiology, The First Affiliated Hospital of Shandong First Medical University, Taian 271016, Shandong, People's Republic of China.; 4 Senior Department of Cardiology, The Sixth Medical Center, Chinese PLA General Hospital, Beijing 100048, People's Republic of China.

**Keywords:** coronary microembolization, regulatory cell death, apoptosis, autophagy, pyroptosis

## Abstract

Coronary microembolization (CME) is defined as atherosclerotic plaque erosion, spontaneous rupture, or rupture of the plaque while undergoing interventional therapy resulting in the formation of tiny emboli that obstruct the coronary microcirculatory system. For percutaneous coronary intervention, CME is a major complication, with a periprocedural incidence of up to 25%. Recent studies have demonstrated that regulatory cell death (RCD) exerts a profound influence on CME through its modulation of inflammatory responses, oxidative stress, cell death, and angiogenesis. RCD, including apoptosis, autophagy, and pyroptosis, is a unique class of genetically highly regulated death patterns pervasive in instances of coronary microembolization. The aim of this review is to summarize the currently known molecular mechanisms underlying CME. Further investigations of the RCD mechanisms may unravel new avenues for the prevention and treatment of CME.

## Introduction

According to a 2020 report from the World Health Organization, about 17.9 million people died from cardiovascular disease in 2019, accounting for about 32% of global mortality 1. Numerous studies have shown that cardiovascular diseases, particularly acute myocardial infarction, are the leading cause of disability and death 2-5. Currently, primary percutaneous coronary intervention is the treatment of choice for AMI 6. The prevalence of coronary microembolization (CME) in primary percutaneous coronary intervention is about 25%, which substantially burdens healthcare resources 7. This is attributed to the rupture of capillaries and bleeding caused by myocardial ischemia-reperfusion following interventional therapy, which promotes the occurrence of CME 8. Currently, there are no effective measures to prevent myocardial ischemia-reperfusion injury. The CME refers to the formation of microemboli that block the coronary microcirculatory system as a result of erosion of atherosclerotic plaque, spontaneous rupture, or rupture of the plaque while undergoing interventional therapy 9, 10. These microemboli have a complex composition, mainly consisting of platelet aggregates, fibrin, hyaluronic acid, and substances from atherosclerotic plaques, including cholesterol 10. A previous report based on a pathological examination of the hearts of 44 patients who experienced sudden death due to coronary heart disease indicated that 89% of the affected vessel calibers from microcirculatory embolism were within 120 μm 11. Of this 89%, 46% were in the range of 40 to 80 μm, while 39% were less than 40 μm 11. Plaque rupture or erosion also leads to the release of soluble pro-thrombotic, vasoconstrictive and pro-inflammatory factors 8. CME induces vasoconstriction and inflammation, which may lead to myocardial contractile dysfunction and myocardial microinfarction, as well as the development of arrhythmias 12. In clinical practice, CME is considered one of the main factors contributing to the no-reflow or slow-flow phenomenon after percutaneous coronary intervention 13. No-reflow or slow-flow is a common complication during percutaneous coronary intervention, characterized by incomplete restoration of blood flow despite successful opening of the coronary vessels, leading to persistent myocardial ischemia symptoms 13. The commonly used clinical treatments (thrombolytic therapy, inhibition of platelet aggregation, and vasodilatation) cannot improve the clinical outcome of CME patients 9. Interestingly, mechanical ischaemic conditioning approaches, involving brief cycles of ischaemia-reperfusion in the heart or a tissue remote from the heart, reduce myocardial infarct size and coronary microvascular damage 8. Although percutaneous coronary intervention with manual thrombus aspiration demonstrated better ST-segment resolution and less distal embolization on angiography compared to primary percutaneous coronary intervention alone, clinical outcome (cardiovascular death, re-infarction, cardiogenic shock, or NYHA class IV heart failure) did not show significant improvement 10. Cardiomyocytes in adult mammals are non-renewable cells. Therefore, the reversal of myocardial damage is crucial for restoring cellular function and preventing cardiomyocyte death 14.

**Figure 1:**
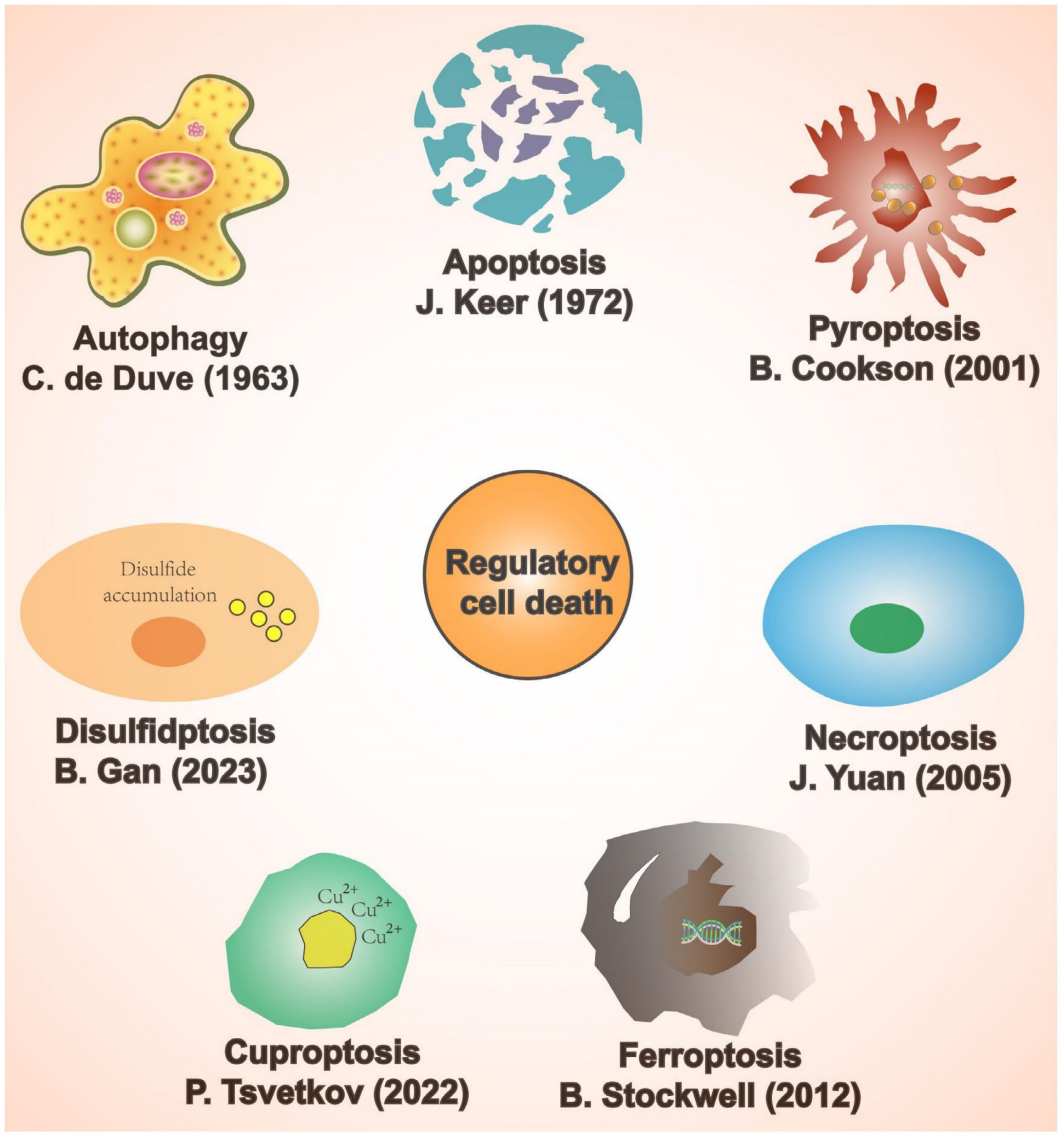
Classification of regulatory cell death.

**Figure 2:**
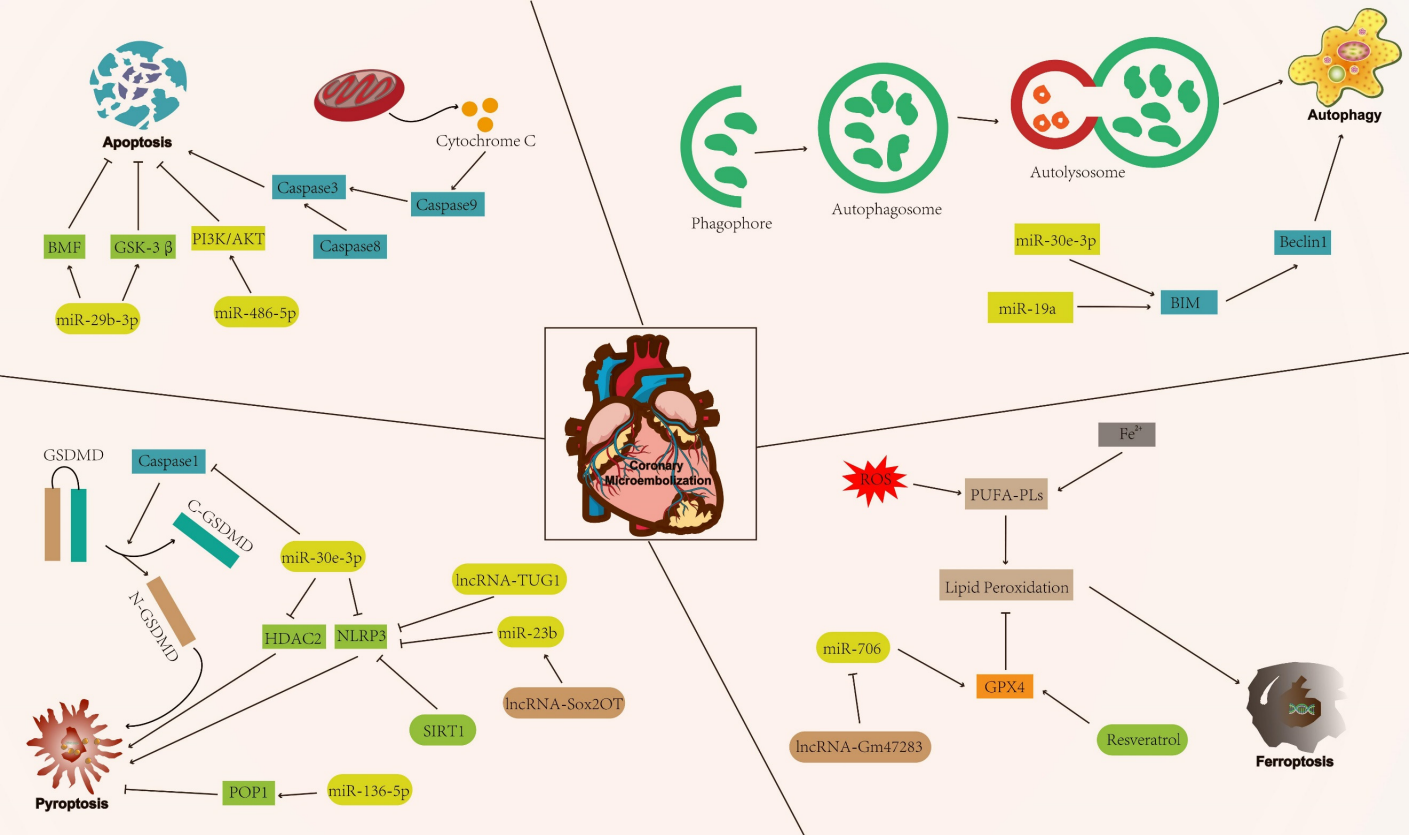
The mechanism of regulatory cell death in coronary microembolization. AKT: protein kinase B; BIM: Bcl-2-like protein 11; BMF: Bcl-2 modifying factor; GPX4: glutathione peroxidase 4; GSK-3β: glycogen synthase kinase 3; PI3K: phosphoinositide 3-kinase; POP1: pyrin only protein 1; SIRT1: sirtuin1.

Cell death patterns include accidental cell death and regulatory cell death (RCD) 15. Accidental cell death is a non-regulated cell death, usually caused by a sudden external injury or stimulus (strong chemicals, radioactive radiation, and physical damage) that exceeds the normal range of cellular response 16. RCD is characterised by a precise molecular mechanism and it is regulated by specific signal transduction pathways. Furthermore, RCD can undergo pharmacological intervention and is regulated by interfering with gene expression and gene-mediated signaling pathways 17, 18. The known forms of RCD include apoptosis, autophagy, pyroptosis, ferroptosis, cuproptosis, disulfidptosis, and necroptosis (Figure 1) 19-25. RCD is closely related to cardiovascular diseases 26. In addition, numerous studies have indicated that RCD plays a significant regulatory role in coronary microembolization (CME) by mediating various signaling pathways involved in its development 27-29. Therefore, precision-targeted therapies can be obtained by modulating the expression of RCD-associated signature genes or their mediated signaling pathways. Although cuproptosis, disulfidptosis, and necroptosis exert a significant influence on the pathogenesis of human disease, they remain understudied in the context of CME. Thus, the aim of this review is to summarize the currently known molecular mechanisms related to RCD (apoptosis, autophagy, pyroptosis, and ferroptosis) in the context of CME (Figure 2).

## RCD in CME

### Apoptosis and CME

Apoptosis is widespread in organisms and is a physiological phenomenon mediated by specific genes 30. Apoptosis is characterized by several key features, including chromatin condensation, cellular shrinkage, DNA fragmentation, formation of apoptotic body, and membrane blebbing 31. Previous study found the presence of apoptosis in the CME model 32. Apoptosis-inducing pathways can be classified into three main categories: mitochondrial, endoplasmic reticulum (ER), and death receptor pathways 33.

The mitochondrial pathway represents a crucial endogenous apoptotic pathway, whereby the activation of the mitochondria-mediated endogenous apoptotic pathway results in a notable reduction in mitochondrial membrane potential, thereby leading to a considerable enhancement in mitochondrial membrane permeability 34. Liu *et al.* 35 showed that the expression levels of ectin-like oxidized low-density lipoprotein receptor-1 (LOX-1), cytochrome c and caspase-9 are significantly increased in a CME model established in Bama miniature pigs. This suggests that CME promotes cardiomyocyte apoptosis and exacerbates CME-induced myocardial injury, possibly through the LOX-1-dependent mitochondrial pathway. A study revealed that rosuvastatin inhibits apoptosis mediated by the mitochondrial pathway and CME-induced cardiac dysfunction in rat CME models by up-regulating B-cell lymphoma-2 (BCL-2) expression and reducing caspase-3, cytochrome c, and BCL-2-associated X protein levels 27. Furthermore, puerarin and resveratrol exhibit comparable efficacy in inhibiting apoptosis and mitigating CME-induced cardiotoxicity, partly due to the increased expression of phosphatidylinositol 3-kinase and protein kinase B in the phosphorylated form 36, 37. MiR-29b-3p expression is significantly reduced in rat CME model 38. Further study showed that miR-29b-3p overexpression mediates neovascularisation, inhibits apoptosis mediated by the mitochondrial pathway, and reduces the area of myocardial microinfarction in rat CME 38. Qin *et al.* 38 demonstrated that miR-29b-3p overexpression mitigates CME-induced myocardial injury, possibly due to the suppression of glycogen synthase kinase 3 and BCL-2 modifying factor (BMF) expression. Moreover, miR-486-5p can mediate the activation of the phosphatidylinositol 3-kinase/protein kinase B axis, thereby attenuating CME-induced cardiomyocyte apoptosis 39. The term "death receptor pathway" is employed to delineate the process by which a cell binds a specific death receptor (Fas or tumour necrosis factor (TNF) receptor) to its ligand (Fas ligand or TNF-α), forming a death signaling complex 40. This complex then initiates a series of intracellular signaling events that ultimately result in apoptosis. Fas-associated death domain (FADD) is an adapter molecule that bridges the interaction between receptor-interacting protein 1 and aspartate-specific caspase-8 41. The caspase-8-mediated death receptor pathway also played an important role in the CME model established in Bama minipigs 35. Notably, TNF-α has been identified as an important causative factor for myocardial contractile dysfunction in CME 7. Leukocyte count and TNF-α contents were increased in the CME posterior myocardium 42. Pretreatment with antibodies to TNF-α appears to prevent contractile dysfunction after CME, whereas in the absence of CME, intracoronary injection of exogenous TNF-α induces contractile dysfunction 42. In conclusion, TNF-α is considered to be an important cause of progressive myocardial contractile dysfunction after CME 42. Zhou *et al.* 43 observed that TNF-α can trigger apoptosis mediated by receptor-interacting protein 1 (RIP1)/FADD/caspase-8 in astrocytes. Furthermore, Su *et al.* 44 indicated that the level of TNF-α is markedly increased in the CME model constructed using Bama miniature pigs. The disruption of the RIP1-FADD complex has been shown to exacerbate myocardial damage 45. However, the effect of TNF-α triggering the RIP1/FADD/caspase-8 signaling pathway on the cardiac in the CME model requires further experimental support. In addition, apoptosis is associated with ER stress, a cellular stress response to the accumulation of unfolded or misfolded proteins in the endoplasmic reticulum lumen 46. The ER-mediated apoptotic pathway has been demonstrated to be an important mechanism of hypoxic injury in cardiomyocytes 47. AMP-activated protein kinase (AMPK) is a key regulatory enzyme involved in energy homeostasis during hypoxia 47. Hypoxia induces activation of the ER-mediated apoptotic pathway in cardiomyocytes, and endogenous activation of AMPK partially reverses these effects 47. In the CME model, targeting the JNK/p38 MAPK pathway was observed to activate the ER stress pathway and induce cardiomyocyte apoptosis, which may be associated with hyperphosphorylation of JNK and p38 48. In conclusion, apoptosis mediates multiple signaling pathways involved in the process of CME.

### Autophagy and CME

Autophagy is a biological process whereby an organism eliminates aberrant proteins or cellular components through the activation of specific genes and their associated signaling pathways, and mainly includes macroautophagy, microautophagy, and chaperone-mediated autophagy 49. Notably, the three forms of autophagy eliminate aberrant cellular components and macromolecules, including proteins, through lysosomes 50. Microautophagy represents a process whereby cytoplasmic carriers are directly phagocytosed through lysosomal membrane invaginations, without the formation of autophagosomes 51. Chaperone-mediated autophagy is the selective degradation of proteins with KFERQ sequences in the cytoplasm via the lysosomal pathway and does not require autophagosome formation 51. A recent study has demonstrated that the activation of chaperone-mediated autophagy provides protection for cardiomyocytes against hypoxic cell death 52. Although microautophagy and chaperone-mediated autophagy have been shown to have important roles in a variety of diseases, their impact in the CME has yet to be extensively studied. Currently, macroautophagy (later referred to as “autophagy” if not otherwise stated) is considered to be the main autophagic branch regulating physiological and pathological mechanisms in the cardiovascular system 53.

Beclin 1, microtubule-associated protein II light chain 3 (LC3-II), and sequestosome 1 are widely employed as indicators to assess autophagy status. Specifically, autophagy activation increases the expression levels of Beclin 1 and LC3-II, while decreasing the levels of sequestosome 1 protein 54-56. Notably, miR-30e-3p expression is elevated under autophagy activation 57. Besides, miR-30e-3p levels are negatively correlated with sequestosome 1 levels in the rat CME model 57. These findings suggest that targeting miR-30e-3p is a promising approach for CME treatment. Moreover, miR-30e-3p directly targets the 3'-UTR of BCL-2-like protein 11 (BIM), decreasing BIM expression, thus activating autophagy and preserving the functional integrity of human-induced pluripotent stem cell-derived cardiomyocytes while mitigating CME-induced cardiac impairment 58. Similarly, miRNA-19a regulates autophagic flux and maintains cardiomyocyte integrity by inhibiting the expression of the pro-apoptotic protein BIM 59. Reduced expression of early growth response factor 1 in the rat model of CME further inhibits BIM expression and up-regulates the level of beclin 1, modulating autophagic flux, thus alleviating CME-induced cardiac impairment 60. Lysosome-associated membrane protein 2a (LAMP2a) protein levels were used as both a primary indicator and driver of CMA function 52. Increased levels of LAMP2a protein were observed in hypoxia-treated cardiomyocytes and in the serum of patients with heart failure 52. In fact, increased levels of LAMP2a protein were thought to be a stress response in cardiomyocytes 52. Furthermore, Ghosh *et al.* showed a significant enhancement of both macroautophagy and chaperone-mediated autophagy activity by increasing LAMP2a protein levels 52. However, the overall effect of the above mechanisms on CME-induced myocardium requires extensive experimental validation. Furthermore, autophagy exerts a dual influence on the regulation of the organism 61. Moderate autophagy is beneficial to the stability of the intracellular environment, while excessive autophagy may lead to cell death, possibly due to basal autophagy in normal cellular activities and induced autophagy under various adverse stimuli 61, 62. Overall, the effects of moderate activation or inhibition of autophagy on the organism should be explored in depth due to the dual effects of autophagy and the complexity of the disease.

### Pyroptosis and CME

The inflammasome is a multi-protein complex essential for regulating the innate immune inflammatory response. NOD-like receptor thermal protein domain associated protein 3 (NLRP3), a member of the NOD-like receptor family, has been extensively studied 63. Pyroptosis is mainly induced by the inflammasome and mediated by gasdermin family proteins 64, 65. Pyroptosis can be divided into classical (caspase-1 mediated) and non-classical (caspase-4, caspase-5, and caspase-11 mediated) pathways 64. In the classical pathway, the inflammasome induces the activation of caspase-1, which specifically cleaves the N-terminal structural domain of gasdermin-D and induces its oligomerization, leading to the disruption of the cell membrane 66. This causes the release of its contents and inflammatory factors (IL-1β, IL-18), ultimately triggering pyroptosis 66. In concrete terms, the NLRP3 inflammasome activates caspase-1, cleaving pro-IL-1β and pro-IL-18 into their active forms, IL-1β and IL-18 67. This triggers pyroptosis and exacerbates the inflammatory response, leading to cardio-depressive effects and cardiac remodeling 68. The non-classical pathway does not require the involvement of inflammatory vesicles but directly activates gasdermin-D via caspase-4/caspase-5/caspase-11, leading to cell membrane rupture and pyroptosis 69.

Recently, NLRP3 inflammasome was proposed as a new biomarker of cardiovascular diseases and predictor of hospitalization and death for myocardial injurie 70. Pyrin domain-containing 1 inhibits excessive NLRP3 inflammasome activity and thereby ameliorates auto-inflammatory disease 71. Pyrin domain-containing 1 regulates the innate immune response by inhibiting nuclear factor-kappa B (NF-κB) transcription factor activity and pro-caspase-1 activation 72. Cai *et al.* 29 demonstrated that miR-136-5p overexpression can increase the level of pyrin domain-containing 1, which inhibits pyroptosis and alleviates CME-induced myocardial injury. Furthermore, miR-30e-3p overexpression reduces the expression of caspase-1 and NLRP3 in the CME rat model 73. Further research showed that miR-30e-3p alleviates CME-induced pyroptosis and inflammatory responses by targeting and inhibiting the expression of histone deacetylase (HDAC) 2, partly due to the reduction of HDAC2 levels, which attenuates the inhibition of mothers against decapentaplegic homolog 7 expression 73. Additionally, miR-142-3p overexpression can target the ataxin 1/HDAC3 axis, promoting the deacetylation modification of histone H3 and inhibiting CME-induced myocardial pyroptosis in rats 74. Zhou *et al.* 75 found that overexpression of lncRNA-taurine up-regulated gene 1 can target the miR-186-5p/x-linked inhibitor of apoptosis protein axis in rat CME models, thus inhibiting NLRP3-mediated pyroptosis and exerting a cardioprotective effect. MiR-200a-3p can also alleviate cardiac dysfunction caused by CME by inhibiting NLRP3-mediated pyroptosis 76. Besides microRNAs, lncRNAs participate in the development of CME-related pyroptosis. LncRNA-Sox2OT can act as a molecular sponge for miR-23b. Also, lncRNA-Sox2OT silencing promotes the binding of miR-23b to the 3'UTR of TLR4 mRNA, thereby inhibiting its downstream NF-κB-mediated signaling pathways, thus alleviating CME-induced cardiomyocyte pyroptosis 77. Besides microRNAs, lncRNAs participate in the development of CME-related pyroptosis. LncRNA-Sox2OT can act as a molecular sponge for miR-23b. Also, lncRNA-Sox2OT silencing promotes the binding of miR-23b to the 3'UTR of TLR4 mRNA, thereby inhibiting its downstream NF-κB-mediated signaling pathways, thus alleviating CME-induced cardiomyocyte pyroptosis 78, 79. Liu *et al.* 80 showed that nicorandil can reduce the expression of thioredoxin-interacting protein and inhibit NLRP3-mediated pyroptosis, thus maintaining the function of rat cardiomyocytes. Additionally, Li *et al.* 81 showed that colchicine can promote the expression of silent information regulator 1 and inhibit NLRP3-mediated cardiomyocyte pyroptosis in rat CME models. Therefore, targeting pyroptosis and related signaling pathways is a potential strategy for CME treatment.

### Ferroptosis and CME

Ferroptosis was first identified by Dixon *et al.* 22 in 2012. Ferroptosis is an iron-dependent cell death that is morphologically and genetically distinct from other RCDs 22. Iron is an essential trace element that mediates various biological processes and maintains the normal life activities of the organism 82. However, excessive accumulation of intracellular Fe^2+^ can contribute to the generation of lipid reactive oxygen radicals and the accumulation of lipid peroxides, which induces ferroptosis 83, 84.

Ferroptosis status is widely detected by measuring intracellular Fe^2+^ concentration, malondialdehyde levels, and the ratio of reduced glutathione/oxidised glutathione 85. Liu *et al.* 86 reported reduced levels of glutathione peroxidase (GPX) 4 and elevated levels of prostaglandin endoperoxide synthase 2, malondialdehyde, and Fe^2+^ by constructing a rat model of CME, suggesting that CME induces the ferroptosis. Further pretreatment of the CME model using desferrioxamine (an inhibitor of ferroptosis) and atorvastatin increased GPX4 expression levels, decreased peroxisomal synthase 2 levels, decreased malondialdehyde and Fe^2+^ levels within the prostaglandins, reduced inflammatory response in the lesion area and significantly improved cardiac function of the rats. Gao *et al.* 87 indicated that miR-706 is a molecular sponge of lncRNA-Gm47283. Furthermore, they showed that knockdown of lncRNA-Gm47283 in the rat myocardial infarction model up-regulates miR-706 levels while decreasing the expression of prostaglandin endoperoxide synthase 2, arachidonic acid 15-lipoxygenase, and GPX4 87. This suggests that lncRNA-Gm47283 knockdown can inhibit ferroptosis and protect cardiac function by targeting miR-706. In addition, resveratrol pretreatment can increase the expression of lysine acetyltransferase 5 and GPX4 in rat myocardial infarction model, suggesting that resveratrol can inhibit cardiomyocyte ferroptosis and alleviate cardiac dysfunction caused by myocardial infarction 88. However, further studies should assess whether lncRNA-Gm47283 and resveratrol exert the same effect of antagonising ferroptosis in the CME model. Ischemia-reperfusion injury is considered a significant cause of CME. Research has confirmed that galangin suppressed ferroptosis through nuclearfactor erythroidderived 2-like 2/ GPX4 signaling pathway activation 89. This suggests that the aforementioned effects may be present in CME. Epidemiological results show that severity of heart disease is related to degree of environmental contamination 90. Di(2-ethylhexyl) phthalate, an environmental pollutant, causes lipid peroxidation and elevated Fe^2+^ levels in cardiomyocytes 90. Further study showed that di(2-ethylhexyl) phthalate induced the onset of ferroptosis in cardiomyocytes by upregulating heme-oxygense-1 90. Obviously, ferroptosis is closely related to the integrity of cardiac function and requires in-depth study in the context of CME.

### Summary and Future Perspectives

In conclusion, RCD is triggered by specific signals that elicit distinct death patterns associated with CME progression. However, the related research has mainly focused on apoptosis, autophagy, pyroptosis and ferroptosis, ignoring other RCD forms, such as cuproptosis, disulfidptosis, and necroptosis. The mechanisms of RCD are multifactorial and complex. Different forms of RCD are associated with distinct characteristic genes and signaling pathways. Furthermore, certain molecular crosstalk occurs between various forms of RCD, which further limits the research. Therefore, a more comprehensive understanding of RCD and CME may facilitate the clinical translation of existing findings. Nonetheless, further studies should comprehensively assess the potential regulatory mechanisms of RCD in CME to provide a definitive reference for the treatment of cardiovascular diseases, including CME.

## Abbreviations

AKT: protein kinase B
BIM: BCL-2-like protein 11
BMF: BCL-2 modifying factor
CME: coronary microembolization
Caspase: cysteine aspartate protease
GPX4: glutathione peroxidase 4
GSK-3β: glycogen synthase kinase 3
HDAC: histone deacetylase
LC3-II: microtubule-associated protein II light chain 3
LOX-1: lipoprotein receptor-1
NLRP3: NOD-like receptor thermal protein domain associated protein 3
NF-κB: nuclear factor-kappa B
PI3K: phosphoinositide 3-kinase
POP1: pyrin only protein 1
RCD: regulatory cell death
SIRT1: sirtuin1
TNF: tumour necrosis factor
